# Does Hypertension affect Saliva Properties?

**DOI:** 10.30476/DENTJODS.2019.80992.0

**Published:** 2020-09

**Authors:** Azra Mohiti, Faeze Eslami, Mohamad Reza Dehestani

**Affiliations:** 1 Dept. of Oral Medicine, Shahid Sadoughi University of Medical Sciences, Yazd, Iran; 2 Student of Shahid Sadoughi University of Medical Sciences, Yazd, Iran; 3 Dept. of Nephrology, Shahid Sadoughi University of Medical Sciences, Yazd, Iran

**Keywords:** Saliva, Blood pressure, Viscosity, Buffer

## Abstract

**Statement of the Problem:**

Systemic conditions can affect the salivary glands and oral health. Hypertension induces xerostomia. Because the function of saliva is related to its quality and quantity, therefore, any changes in saliva can lead to diminished quality of patient’s life.

**Purpose:**

The aim of this study was to determine the relationship between pH and viscosity of cumulative unstimulated saliva and hypertension in adults with sustained hypertension.

**Materials and Method:**

This cross sectional study took place on patients referred to oral medicine faculty of Shahid Sadoughi University of Medical Science. The patients’ blood pressure was measured and the 135 patients fitting the inclusion criteria participated in the study. Their unstimulated cumulative saliva was collected by spitting method and pH of the samples was measured by digital pH-meter set. The viscosity of the samples was measured by comparing the amount of saliva displacement in the thistle tube with control fluids at mm/10 seconds. The data was analyzed by SPSS version 20 software and ANOVA tests and Tukey multiple comparison and their nonparametric equivalent (*p*≤ 0.005).

**Results:**

The results of this study showed that there was a significant relationship between pH and viscosity of unstimulated saliva of normotensive and borderline hypertensive patients (*p*<.0001and *p*< .005, respectively) and between normotensive and stage I hypertensive patients (*p*<.0001, *p*<0.000). Therefore, hypertension had a direct and significant relationship with saliva viscosity but a reverse relationship with saliva pH.

**Conclusion:**

Hypertension can reduce the pH and increase the salivary viscosity in hypertensive patients, which subsequently lead to changes in quality and quantity of secreted saliva and influence the oral health and quality of the patient’s life.

## Introduction

High blood pressure (BP) or hypertension is one of the many challenges among elderly people; most of them need to take antihypertensive medications. Stabled systolic BP ≥140 mmHg and/ or a stabled diastolic BP ≥90 mmHg are defined as hypertension in adults. High BP can gradually lead to higher morbidity and mortality rates because of cardio vascular diseases [ 1 ]. Saliva is the most important oral fluid and saliva pH and viscosity are among the most important properties of saliva, which have not been studied fully. Systemic diseases and every day medications used by the patient can cause change the quantity and quality of secreted saliva by salivary glands, effecting the patient’s quality of life [ 2 , 5 ].

The viscosity of secreted saliva is dependent on many factors such as its protein content or inorganic part. Any disturbances in saliva viscosity can indicate the changes in saliva content and may have an impact on the health and integrity of oral mucosa. In addition, saliva pH is one of the most important features of saliva and is directly correlated to the buffering capacity of secreted saliva, and plays a significance role in mai-ntaining oral dental health and dental integrity [ 6 , 7].

The prevalence of hypertension is increased by age [ 1 ] and many studies have been conducted on this problem but there is little information about the influence of hypertension and its treatments on saliva properties and the influence on salivary gland function. Since high BP has many effects on body fluids including saliva, these effects need to be determined [ 2 , 8 ]. Thus, the aim of this study was to evaluate the relationship between hypertension and pH and viscosity of cumulative saliva in adults with hypertension.

## Materials and Method

This cross sectional study was conducted on patients referred to Oral Medicine Faculty of Shahid Sadoughi University of Medical Sciences. The Ethical Committee of Shahid Sadoughi University of Medical Sciences of Yazd (No ir.ssu.rec.1395.128) approved this study. 

The medical history of participants were obtained by interview and recorded in their files. Using sequential sampling, 135 patients were included in this study after receiving a written consent form from all participants. According to their BP, they were divided in to 3 groups with 45 participates in each. All groups were matched for age and gender.

The inclusion criteria for participants were just hypertensive individuals with no other systemic disease, taking no other medications (except antihypertensive medications such as losartan, beta-blockers, and so on) and no history of smoking or alcohol use. Patients who were taking diuretics for their hypertension or taking medications, which are known to induce xerostomia such as antidepressants, were excluded from the study. 

BP was taken twice (by 5 minutes intervals) after each individual was seated comfortably on the chair for at least 5 minutes, by SANA automatic Sphygmomanometer (model HL868RT, made by Health and life Company in Taiwan). To reduce inter- examiner error, automatic Sphygmomanometer was used.

Hypertension was defined as systolic BP more than 140 mmHg and diastolic BP more than 90 mmHg. The participants were divided into 3 even groups. Group 1 consisted of 45 normotensive individuals (BP less than 120/80 mmHg, not taking medications), group 2 consisted of 45 borderline hypertensive individuals (systolic BP in range of 120-139 or diastolic BP in range of 80-89 mmHg, not taking medications), and group 3 consisted of 45 stage I hypertensive individuals (systolic BP in range of 140-159 or diastolic BP in range of 90-99 mmHg). Spitting method was used to collect the unstimulated whole saliva. All of the saliva samples were collected at 25 degrees C. at 9-11 A.M. Individuals were forbidden to eat, drink, smoke, or brush their teeth, at least 90 minutes before sampling in order to decrease the influence of daily changes on the composition of saliva.

Before sampling, participants were remained seated on the chair and asked to swallow all the saliva in their mouth. Then, they were asked not to swallow their saliva for 5 minutes and spit the collected saliva into the sterilized cups provided by the investigators.

Immediately after sampling, pH of the saliva samples were measured by Pen-type digital pH-meter set (AZ Company, Taiwan). In addition, the viscosity of samples was immediately measured by comparing the amount of displacement of saliva in the calibrated thistle tube to that of control fluids. Control fluids were glycerin (Viscosity=830 mm^2/s (cSt)) and water (Viscosity=1 mm^2/s (cSt)) and the amount of displacement of the fluid in the thistle tube was compared to the control fluids within 10 seconds (mm/10 s) in this study. According to Shapiro test, data distribution was normal and ANOVA test was used to compare the groups. When the differences were significant, Tukey multiple comparisons and/or nonparametric equivalent was used to compare groups pair wise. The *p*< 0.05 was regarded significant.

## Results

In this study, 51% of individuals were male and 49%were females. In addition, 16% of individuals were taking antihypertensive medications including 82% took Losartan and 18% propranolol. Furthermore, 76% of individuals took only one medication and 24% took two. All groups were matched regarding gender and age. The mean ages of the 3 groups were 45.59 years for group 1 (SD:12.08), 45.53 years for group 2 (SD:9.91) and 46.56 years (SD:13.40) for group 3. The mean and standard deviation of salivary pH of three groups are compared in Table 1. As shown in Table 1 and Table 2, the average pH was higher in the group 1 than that in group 2 (*p*=.0001) and it was higher in group 2 than in group 3 (*p*=.005). These differences were statistically significant. As shown in Tables 3 and 4, the average of displacement of saliva in the thistle tube at mm/10s in group1 was more than group 2 (*p*=.005) and greater in the group 2 than that in group 3 (*p*=.0001). These differences were statistically significant (*p*< .05).

## Discussion 

The results of this study demonstrated the relationship between hypertension, antihypertensive medications, and their influence on saliva properties. The participants involved in this study were only hypertensive individuals without any other systemic diseases and a few of them took particular medications for their high BP (such as losartan, beta-blockers) but not diuretics because it has been shown that diuretics cause xerostomia [ 1, 2].

**Table 1:** The mean and standard deviation of salivary pH in 3 groups (n = 135).

Groups	Std.Deviation	Mean	Number
Group 1 (Normal BP)	0.42628	6.487	45
Group 2 (Pre-hypertension)	0.50526	6.060	45
Group 3 (Stage I hypertension)	0.56959	5.851	45

ANOVA Test *p*= 0.000

**Table 2:** Multiple comparisons of salivary pH in 3 groups.

Groups	Mean Difference	*p* Value
Group 1 (Normal BP)	0.426 Group 2	0.0001
	0.636 Group 3	0.0001
Group 2 (Pre-hypertension)	0.426 Group 1	0.0001
	0.209 Group 3	0.151
Group 3 (Stage I hypertension)	0.636 Group 1	0.0001
	0.209 Group 2	0.151

**Table 3:** The mean and standard deviation of saliva displacement in thistle tube (mm/10s) amongst 3 groups.

Groups	Std.Deviation	Mean	Number
Group 1 (Normal BP)	2.6310	4.678	45
Group 2 (Pre-hypertension)	1.9932	3.233	45
Group 3(Stage I hypertension)	1.6963	2.556	45

ANOVA Test, *p*= 0.0001

**Table 4:** Multiple comparisons of the saliva displacement in thistle tube amongst 3 groups.

Groups	Mean Difference	*p* Value
Group 1 (Normal BP)	1.444 Group 2	0.005
	2.122 Group3	0.0001
Group 2 (Pre-hypertension)	1.444 Group1	0.005
	0.677 Group3	0.408
Group 3 (Stage I hypertension)	2.122 Group1	0.0001
	0.677 Group2	0.408

BP was measured twice for a more powerful prediction. To remove the differences among examiners, an automatic sphygmomanometer was employed.

Unfortunately, a few studies have been done on the effects of hypertension on saliva. In a study, R. Kagawa et al. [ 1 ] found that pH of unstimulated saliva is significantly lower in hypertensive individuals. In fact, their results showed that the increase in both systolic and diastolic BPs could lead to decrease in pH of unstimulated saliva [ 1 ]. The results of current study showed that the decrease in saliva pH and hypertension are related; this finding is similar to R. Kagawa’s [ 1 ] results. Our results showed that pH of unstimulated saliva in stage I hypertensive individuals and borderline participants was lower than those with normal BP, therefore, a reduction in pH can cause changes in physical and chemical properties of saliva , which subsequently may influence the oral health [ 9 , 10 ].

Preoteasa et al. [ 11 ] expressed that viscosity and pH of saliva are both independent parameters and their changes are not related to each other; while pH decreases, viscosity tends to increase. This is consistent with our results, as we also did not find any relationship between saliva viscosity and pH in the participants in our study.

The viscosity of samples was measured just immediately after collection due to the possibility of its rapid changes by time. As mentioned in previous studies, temperature can negatively influence the viscosity of saliva; therefore, in the current study the amount of sample displacements in thistle tube (mm/s) was measured at 25oC to prevent the effect of temperature [ 12 ]. The statistical analysis of this study showed that, the amount of salivary displacement in the thistle tube in stage I and pre hypertensive groups was significantly lower than normotensive group. Therefore, the viscosity value was higher in hypertensive groups and because of higher viscosity, saliva travels less in the thistle tube in the hypertensive groups. It can be inferred that by increasing BP, the viscosity of unstimulated saliva also increases resulting in lower salivary flow. This would consequently influence the cleansing effect of saliva on teeth and oral mucosa resulting in impaired oral health in these individuals [ 14 ].

In addition, pH of samples was measured immediately after collection because pH of saliva increases while it is exposed to the air due to constant losing of CO_2_ [ 4 ]. The findings of current study showed that pH of unstimulated saliva in stage I hypertensive and pre hypertensive groups was significantly lower than in normotensive group. It may be suggested that the decreased salivary pH in patients with hypertension could be related to the reduction of unstimulated salivary flow rate in these patients before taking medications. This might be due to higher activity in the sympathetic pathways and lower activity in the parasympathetic pathways, which controls the salivary secretion and leads to lower salivary flow rate [ 5 ]. Moreover, bicarbonate (the most important oral buffer) is more effective in higher salivary flow rates and in lower flow rates its concentration decreases extremely, resulting in lower pH, and buffering capacity of the saliva [ 2 ]. It could be stated that, pH of saliva is dependent on the amount of secreted saliva and the speed which is secreted by the salivary glands, therefore, influencing the components of the saliva. Besides, because of decreased saliva secretion in high BP patients, pH is reduced and buffering capacity is compromised [ 11 , 15 , 16 ]. The reason for this increase in bicarbonate concentration is the elevation of saliva secretion rate, and in general, bicarbonate concentration is low in all salivary glands. However, when metabolic activity is increased, CO_2_ is produced and is hydrated by carbonic anhydrase enzyme, therefore, lower saliva secretion results in lower bicarbonate concentrations [ 17 ]. Salivary buffering capacity has a great influence on pH of plaque surrounding the enamel and plays an important role in prevention of dental caries progression [ 18 ]. Therefore, in individuals with high BP, the concentration of bicarbonate and consequently its buffering capacity diminishes because salivary flow rate and saliva pH is reduced in these patients. If this reduced pH in oral cavity is sustained for a long period, it can lead to colonization of non- useful bacteria, which leads to higher caries rate compared to normotensive individuals and the oral health of the patient is compromised [ 19 ]. It is reported that salivary viscosity is related to salivary protein content [ 8 ]; therefore, any alterations in protein secretion of saliva modifies its properties and these cha-nges lead to irreversible complications in oral health [ 13 ]. Lubrication of mouth, larynx, and other soft and hard tissues is one of the most important roles of saliva, which is made by elastic resistance between surfaces, and viscosity is the most important indicator of saliva lubricating role [ 14 ]. Consequently, increased viscosity results in rampant caries, oral mucositis, difficulty in swallowing, halitosis, and the early tooth loss due to caries, which all these consequences may influence the life quality of a patient with hypertension [ 20 , 22 ]. It seems reasonable to monitor chronic hypertensive patients that take antihypertensive medications and have a history of root caries or periodontal diseases more frequently [ 16]. 

In this study, one of our limitations was the small sample size of patients who were taking medication in stage I hypertension; therefore, we could not compare subjects taking medication with those who did not. However, for reducing the effect of medication, patients taking medications with known effects on BP were excluded from the study. Future studies are suggested to be done only on patients with stage II hypertension who are taking the same medication for better results.

## Conclusion 

Hypertension can bring about reduced pH and increased salivary viscosity in hypertensive patients, which subsequently leads to changes in quality and quantity of secreted saliva and influences the oral health and quality of the patient’s life. 
